# Cortisol Response to Psychosocial Stress in Chinese Early Puberty Girls: Possible Role of Depressive Symptoms

**DOI:** 10.1155/2015/781241

**Published:** 2015-06-04

**Authors:** Ying Sun, Fang Deng, Yang Liu, Fang-Biao Tao

**Affiliations:** ^1^Department of Maternal, Child & Adolescent Health, School of Public Health, Anhui Medical University, Hefei, Anhui 230032, China; ^2^Anhui Provincial Key Laboratory of Population Health & Aristogenics, Hefei, Anhui 230032, China

## Abstract

*Objective*. The present study aimed at investigating unique patterns of salivary cortisol reactivity and recovery in response to a social stressor among girls with early puberty and exploring possible role of depressive symptom in this association. *Design*. Case-control study. *Patients*. Fifty-six girls with early puberty and age- and body mass index- (BMI-) matched normal puberty controls (*n* = 56) were selected. *Measurements*. Salivary cortisol was measured in response to the Groningen Social Stress Test for Children. *Results*. Girls with early puberty had higher cortisol concentration at the end of the GSST (C3), cortisol concentration 20 min after the end of the GSST (C4), and AUC increment (AUCi) compared to non-early puberty girls. Depressive symptoms correlated with blunted HPA reactivity among girls with early puberty. *Conclusion*. This study demonstrated the disturbance effect of objectively examined early pubertal timing on HPA axis responses. It also suggested that stress reactivity might be blunted for individuals with depressive symptoms.

## 1. Introduction

Animal studies indicate that there are substantial prepubertal- and adolescent-related changes in the stress reactivity of the HPA axis [1]. Cortisol reactivity has emerged as an important marker of stress responsivity and a key component of risk for symptoms of psychopathology in youth. Additional research is needed examining cortisol reactivity within populations at risk for symptoms of psychopathology (e.g., children with early puberty). To date, there is few studies report of cortisol reactivity in children classified as early puberty. Sontag-Padilla et al. [2, 3] have explored cortisol reactivity among girls who had evidence of premature adrenarche (PA) and girls with early menarche or parent-report early pubertal development. However, PA is the early activation of the HPA axis through which the concentrations of adrenal androgens increase beyond what is usually seen in age-matched peers, and the average age of subjects was 11.84 ± 0.77 years, which was about 2~3 years after pubertal onset. Thus, it is necessary to explore HPA axis reactivity in response to social stresses among early pubertal onset children determined by early thelarche through physical examination.

The present study investigated unique patterns of salivary cortisol reactivity and recovery in response to a social stressor among girls with early puberty. We proposed that early pubertal timing may have a lasting influence on HPA axis reactivity to social stress. Specifically, early maturing girls would show great reactivity to social stress compared to on-time maturing girls.

Findings regarding association between cortisol stress responsivity and depressive symptoms are less consistent. Both elevated and blunted levels of cortisol secretion during childhood and adolescence have been linked to the subsequent onset of major depressive disorder (MDD) [4]. In the study, therefore, we examined the influence of depressive symptoms on HPA axis reactivity to social stress among girls with and without early puberty.

## 2. Methods

### 2.1. Participants

All the children came from an urban school-based follow-up study. Parents provided active consent; written assent was obtained from participants themselves. After examination of height, weight, and pubertal status (assessed by inspection of the breasts for girls by one female endocrinologist) of all students, fifty-six girls with early puberty from grade 1 to grade 3 were selected as the case group. The age- and body mass index- (BMI-) matched normal puberty controls (*n* = 56) were recruited from the same class.

### 2.2. Measures

#### 2.2.1. The Groningen Social Stress Test

Experimental sessions took place on weekdays, in sound-proof rooms with blinded windows at selected locations in the participants' school; lasted about 2~3 hours; and started between 14:00 h and 15:30 h. At the start of the session, the test assistant, blind to the participants' pubertal timing, explained the procedure. Participants were, on the spot, instructed to prepare a 6 min speech about themselves and their lives and deliver this speech in front of a video camera. After the 6 min speech, participants were instructed to subtract 17 repeatedly, starting with 13278. Details of the experimental procedure and preparation of GSST have been previously described [5].

#### 2.2.2. Cortisol Sampling during the GSST

HPA axis responses toward the GSST were assessed by four salivary samples of cortisol, referred to as C2 to C5. The protocol began with a 10 min rest period to allow the participant to adjust to the research setting. Participants then provided a baseline saliva sample (C1). They were then led into the experimental room and introduced to two research assistants.

Sample C2 was collected before the GSST, reflecting pretest HPA axis activity during rest. At that time, participants were filling out rating scales while sitting quietly. C3 was collected immediately after the GSST, reflecting HPA axis activity at the beginning of the GSST, when participants had to deliver a speech. C4 was collected 20 min after the end of the GSST, reflecting HPA axis activity at the end of the GSST. Finally, C5 was collected 40 min after the end of the GSST, reflecting poststress HPA axis activity.

#### 2.2.3. Pubertal Timing

Pubertal breast Tanner stage of each girl was assessed by the same female pediatric endocrinologist. Tanner staging was done by palpation of breast tissue in addition to visual inspection. Pubertal timing was operationalized as whether level of breast Tanner development of a girl was more or less advanced than that of matched peers at time of the study. In this study, the 25th percentile age of B2 and B3 was used as the cut-off points of early pubertal timing [6], which is 8.0 y and 9.8 y, respectively, according to China Puberty Collaboration Study [7]. Subjects who met criteria, (i) onset of breast development (B2) <8.0 years of age or (ii) enlargement of breast tissue (B3) <9.8 years of age, were classified as early puberty.

#### 2.2.4. Depressive Symptoms

The Short Mood and Feelings Questionnaire Child Version (SMFQ-C) [8] was administered by face-to-face interviews with each child after the test of GSST. SMFQ-C is a brief (13-item) questionnaire enquiring about the occurrence of depressive symptoms over the past 2 weeks. The 90th percentile of SMFQ-C scores is 7 among all the 7~9-year-old girls investigated in grade 1 to grade 3.

### 2.3. Biochemical Analyses

#### 2.3.1. Cortisol Sample

Salivary cortisol samples were collected using Salivettes, which are small cotton swabs in plastic tubes (Sarstedt, Nümbrecht, Germany). After the experimental session, the samples were placed in a refrigerator at −4°C and within three to four days brought to the laboratory of the School of Public Health, Anhui Medical University, in Hefei and stored at −20°C until analysis. All samples were analyzed with the same reagent, and all experimental samples from a participant were assayed in the same batch. Missing experimental samples (C1, *n* = 1; C2, *n* = 2; C3, *n* = 2; C4, *n* = 3; C5, *n* = 3) were due to detection failures in the lab (36.4%) or insufficient saliva in the tubes (63.6%). Missing values were imputed on the basis of a combination of the group mean and standard deviation for the missing cortisol sample and the mean of the participant's cortisol samples that were present.

#### 2.3.2. Cortisol Parameters

Because we were particularly interested in the HPA axis response to stress, we used the area under the curve with respect to the increase (AUCi) as an outcome measure. The AUCi represents the area under the curve above baseline levels (cortisol sample C2). It was computed according to the method described elsewhere [9].  Table 1 introduces means and standard deviation of the variables used in the study.

### 2.4. Statistical Analysis

Means of age, BMI, and four salivary cortisol samples during the Groningen Social Stress Test (GSST) were compared in early puberty and normal puberty girls by the Student *t*-test. Chi-square test was used to compare occurrence of depressive symptoms among the two groups. ANOVA test was used to test differences in AUCi between early puberty and normal puberty girls with or without depressive symptoms followed by LSD (Least Significant Difference) post hoc test.

### 2.5. Ethical Considerations

The study is approved by the ethical committee of Anhui Medical University. All children and parents received written information and were invited to an information meeting. The study is presented as a study on growth and puberty timing. All participants and their parents gave informed consent.

## 3. Results

Descriptive statistics are given in Table 1, stratified by pubertal timing. Adolescents in the early puberty and control groups did not differ regarding age, BMI, C2, and C5 during GSST session. Girls with early puberty had higher C1, C3, C4, and AUCi compared to non-early puberty girls.

Mean cortisol concentrations of the participants were lowest at 40 min after the end of the GSST (sample C5), whereas they were highest at the end of the GSST (sample C4). Cortisol response to the GSST, as indicated by the mean cortisol concentration of every sample, was higher in early puberty compared with control girls.

**Figure 1:**
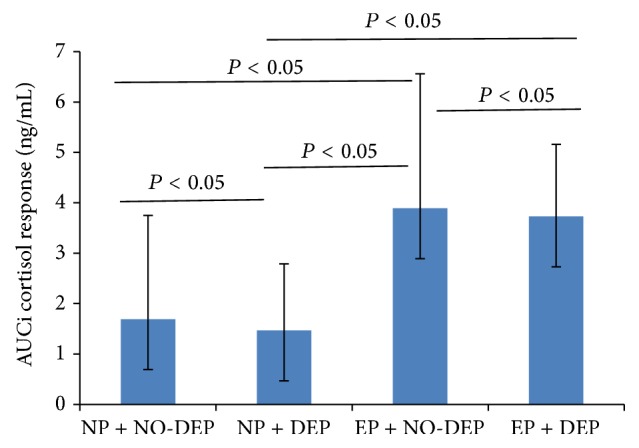
AUCi of the cortisol response to the GSST, according to pubertal timing and depressive symptoms. AUCi is short for area under the curve with respect to the increase. EP is short for early puberty; DEP for depressive symptoms; NP for normal puberty; NO-DEP for no depressive symptoms.

**Figure 2:**
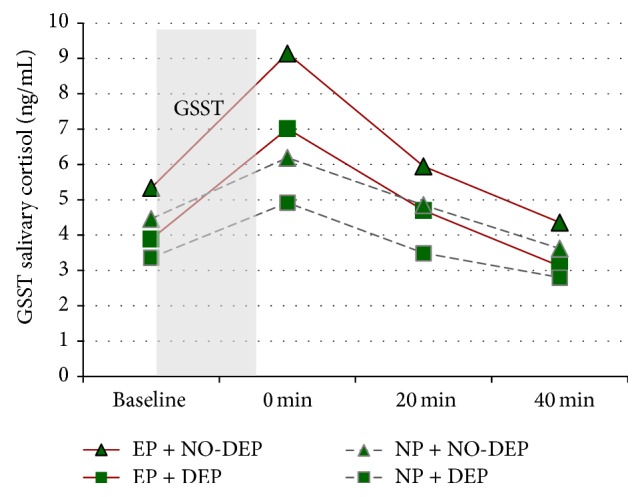
Salivary cortisol responses to GSST by pubertal timing and depressive symptoms. EP is short for early puberty; DEP for depressive symptoms; NP for normal puberty; NO-DEP for no depressive symptoms.

Figures 1 and 2 indicated that, in early puberty group and normal puberty group, AUC increment was lower in girls with depressive symptoms. Girls with early puberty and no depressive symptoms had the highest AUC increment in salivary cortisol in response to stress. Girls with normal puberty and depressive symptoms had the lowest AUC increment in salivary cortisol in response to stress.

## 4. Discussion

In the present study, we examined the relationship between early puberty and HPA axis response to psychosocial stress in adolescent girls. As hypothesized, we found that early puberty was related to an increased cortisol in response to a social stress. It is also found that stress reactivity was blunted for girls with depressive symptoms. This study is the first to demonstrate the disturbance effect of objectively examined early pubertal timing on HPA axis responses.

Recent human and nonhuman animal studies have shown that hormonal stress reactivity increases significantly throughout puberty and adolescence [1, 10]. Only a few cross-sectional studies have investigated the effects of puberty on the cortisol responses to a public performance task between late childhood and adolescence. van den Bos et al. [10] first demonstrated an increased overall response of the HPA axis (cortisol) to a public speaking task with self-reported pubertal development. A limitation of this study is that they used a self-report method of pubertal development rather than examination by trained physicians. Although this study found positive relations between self-reported pubertal development and the responses of the HPA axis to a social-evaluative situation, stronger results may be obtained using physician ratings of pubertal development, as these are more reliable. Gunnar et al. [11] compared the cortisol response to the TSST for 9-, 11-, 13-, and 15-year-old girls. They observed a larger cortisol response in 15-year-old than in 11-year-old girls. Moreover, there was a marginally significant correlation between the cortisol response and pubertal stage as measured by self-report on the Pubertal Development Scale. Smith and Powers [12] investigated associations between retrospectively assessed timing of pubertal development and HPA axis reactivity to an interpersonal stress task in 110 young adult women. The results showed that, for earlier developing girls, higher levels of interpersonal conflict were associated with greater physiological stress in anticipation of the discussion task and less physiological recovery following the discussion. But the retrospective assessments of pubertal timing and study population made it remain unclear about possible unique effects on HPA axis function of early pubertal timing during adolescence.

Pubertal timing in this study was determined by breast Tanner stage through physician examination in young girls. Considering most of those girls do not have pubertal onset, it is reliable that those classified as early puberty truly matured earlier than those controls. The findings support the hypothesis that early puberty could interfere with HPA axis function and increase sensitivity to social stress among early maturing girls.

Because the HPA axis is sensitive to gonadal hormone-dependent organization during critical periods of development, it is proposed that [13] exposure to the pubertal rise in gonadal hormones to some extent organizes the function of the HPA axis. Given that gonadal hormone levels are substantially different in early puberty and normal puberty girls, we assume that differences in gonadal hormones were at least in part responsible for the shifts in stress reactivity between the two groups.

Interestingly, it is worth noting that girls with depressive symptoms in early puberty group showed lower increases in AUC increment in response to stress compared with girls without depressive symptoms, which suggests that stress reactivity was blunted for individuals with depressive symptoms. This is consistent with a lately published study which showed that the onset of MDD was predicted by cortisol hyporeactivity in girls who were earlier in pubertal development [14]. The authors proposed that both elevated stress reactivity and blunted cortisol secretion appear to be risk factors for the development of depression. This aberrant responding of the HPA axis may reflect difficulty in regulating emotional responses in a stressful context, which might in turn place adolescents at risk for developing emotional disorder including depression. Dysregulation of the HPA axis can take the form of hypoactivity or reactivity, a pattern of dysregulation that has been associated with distress disorders. These data might support the hypothesis that exposure to depression symptoms may lead to alterations in patterns of stress reactivity as has been found in many studies [15, 16].

It is important to note that, however, although GSST provoked a heightened cortisol response in early puberty girls, salivary cortisol levels before GSST (C2) and 40 min after GSST (C5) did not differ between early pubertal and control girls. We propose that the speed of recovery of the HPA axis after its activation by stressor in early puberty girls is still sensitive.

Future research should focus on HPA axis function over time with the variation of pubertal timing through longitudinal follow-up, especially those originally classified as early pubertal timing and later becoming on-time pubertal timing.

Our findings, however, should be interpreted in light of the limitations of this study. First, this was a cross-sectional study, so we could only discriminate between currently depressed and nondepressed girls. There is evidence from several neuroimaging studies which suggests that persistent depression coincides with changes in the brain that might affect HPA axis functioning [16]. Dysregulation of the functioning of the HPA axis develops following repeated experience of depression symptoms. Much more evidence is needed on the developmental interface of HPA axis functioning and depression by testing associations between repeated measures of depression symptoms and HPA axis reactivity throughout adolescence.

Second, basal HPA-activity and measures of the diurnal circadian cortisol rhythm, for example, cortisol awakening response (CAR) with wake-up cortisol, bedtime cortisol, diurnal slope, and total cortisol area under the curve (AUC), were not assessed in our study.

Finally, although the Groningen Social Stress Test (GSST) is a standardized protocol for the induction of moderate performance-related social stress, employment of GSST makes it difficult to compare our results to other studies using Trier Social Stress Test-Child Version (TSST-C) as psychological stress challenges. Thus, our conclusions regarding the AUCi in GSST should be interpreted with some caution as we only had four time points on which our calculation was based.

## 5. Conclusion

In summary, results from the present study indicate that early puberty is associated with hyperreactivity of HPA axis in response to social stress. The study also showed blunted HPA axis reactivity among girls with depressive symptoms during the onset of puberty. Incorporating more HPA axis activity measures into on-going population-based, longitudinal studies will help to expand our understanding of the role of the HPA axis in the association of early puberty with psychopathological outcomes.

## Figures and Tables

**Table 1:** Means (standard deviation) and report rate of the variables used in the study.

Variable	Mean (SD)	Early puberty	Control	*P*
Age (years)	8.21 (0.51)	8.21 (0.47)	8.22 (0.55)	>0.05
BMI	17.04 (2.65)	16.94 (2.80)	17.14 (2.51)	>0.05
Depressive symptoms	28.6%	38.2%	19.3%	0.036
Baseline cortisol				
C1 (ng/mL)	5.80 (3.04)	6.44 (3.18)	5.18 (2.80)	0.029
GSST cortisol				
C2 (ng/mL)	4.79 (2.40)	4.80 (1.58)	4.26 (1.73)	>0.05
C3 (ng/mL)	6.51 (3.13)	8.31 (2.29)	5.96 (1.75)	<0.001
C4 (ng/mL)	4.95 (2.38)	5.48 (1.86)	4.61 (1.76)	0.013
C5 (ng/mL)	3.81 (1.82)	3.89 (1.44)	3.47 (1.46)	>0.05
AUCi (ng/mL)	1.41 (2.51)	4.07 (1.691)	1.60 (1.79)	<0.001

C1 = baseline cortisol; C2 = before GSST; C3 = cortisol concentration at the end of the GSST; C4 = cortisol concentration 20 min after the end of the GSST; C5 = cortisol concentration 40 min after the end of the GSST; AUCi = area under the curve with respect to the increase.
